# TRPM7 and MagT1 in the osteogenic differentiation of human mesenchymal stem cells in vitro

**DOI:** 10.1038/s41598-018-34324-8

**Published:** 2018-11-01

**Authors:** Sara Castiglioni, Valentina Romeo, Laura Locatelli, Alessandra Cazzaniga, Jeanette A. M. Maier

**Affiliations:** Dipartimento di Scienze Biomediche e Cliniche L. Sacco, Università di Milano, Milano, I-20157 Italy

## Abstract

Mesenchymal stem cells are fundamental for bone formation and repair since they respond to microenvironmental stimuli by undergoing osteogenic differentiation. We show that the kinase and cation channel TRPM7 and the magnesium transporter MagT1 have a role in harmonizing the osteogenic differentiation of human mesenchymal stem cells. TRPM7 and MagT1 are upregulated in osteogenic differentiation and silencing either one accelerates osteogenic differentiation, partly through the activation of autophagy. Intriguingly, similar results were obtained when the cells were cultured under magnesium deficient conditions. These results underpin the contribution of magnesium, TRPM7 and MagT1 to autophagy and osteoblastogenesis.

## Introduction

The bone is a metabolically active tissue that is continuously remodeled in development and throughout life to repair microdamages and adjust its architecture to changing mechanical needs^1^. This dynamic process relies on the coordinated and timely balance between bone resorption by osteoclasts and bone formation by osteoblasts. In particular, osteoblasts arise from bone marrow mesenchymal stem cells (MSC), rare pluripotent cells that activate the genetic program leading to osteoblastogenesis in response to specific stimuli from the microenvironment^2^. There is a growing interest in MSC because of their use in cell-based therapy as a treatment strategy in orthopedics. It is therefore essential to disclose the molecular events involved in their differentiation into osteoblasts. Both chemical and physical cues modulate the fate commitment of bone MSC^3^. In particular, upon exposure to shear forces MSC exhibit dose- and time-dependent changes in gene expression that lead to the acquisition of an osteogenic phenotype^4^. Recently, Transient Receptor Potential Melastatin 7 (TRPM7), a dual-function kinase and cation channel, has been shown to mediate the osteogenic differentiation of murine MSC in response to shear stress^5^. Accordingly, in these cells TRPM7 directly senses membrane tension and is involved in mechanotransduction^6^. Moreover, TRPM7 is fundamental for murine MSC survival^7^. While TRPM7 is implicated in the transport of divalent cations, primarily calcium (Ca) and magnesium (Mg)^8^ both crucial components of the bone, Mg transporter 1 (MagT1), which is expressed in all human tissues, selectively transports Mg across the plasma membrane^9^. Rather little is known about the expression and the role of MagT1 in bone. Rat MSC cultured on Zn/Mg surfaces, which promote osteogenesis, significantly upregulate MagT1 gene expression^10^. In rat MSC, silencing *MagT1* blunts osteogenic differentiation^11^. Since both MagT1 and TRPM7 contribute to the maintenance of Mg homeostasis at the cellular level, it should be recalled that Mg, the fourth most abundant metal ion in the body mostly stored in the skeleton^12^, plays a crucial role in bone metabolism and in the regulation of bone cell functions^13^. A recent report shows that Mg deprivation as well as mesendogen, an inhibitor of TRPM7, robustly enhance mesoderm and definitive endoderm differentiation of embryonic stem cells^14^.

On these bases, we investigated the expression and the role of *TRPM7* and *MagT1* in human MSC (hMSC) induced to differentiate into osteoblasts by exposure to an osteogenic cocktail. We evaluated the expression of some osteogenic differentiation markers. In particular, we focused on Runt-related transcription factor 2 (*RUNX2*) and collagen type I (*COL1A1*). *RUNX2*, the master regulator of osteogenesis^15^, acts early to commit MSC to the osteochondral lineages and then induces the expression of *COL1A1*, which is crucial for the osteogenic phenotype. The rational for studying the expression of *RUNX2* and *COL1A1* resides in our recent findings showing that if the upregulation of *RUNX2* is not accompanied by the increase of *COL1A1*, calcium deposition does not occur and this prevents hMSC full differentiation into osteoblasts^16^.

A connection exists between osteogenesis and autophagy, an evolutionary conserved self-degradative system that delivers cytoplasmic constituents to the lysosomes^17^. *In vivo*, the deletion of FIP200, an essential component of the autophagosome complex, suppresses autophagy and causes osteopenia in mice by inhibiting osteoblast differentiation^18^. Similarly, knocking down autophagy-related gene *ATG5*, a component of the autophagosome, reduces bone mineralization^19^. *In vitro*, the autophagy proteins ATG7 and beclin 1 are required for mineralization in an osteoblastic cell line^19^. Therefore, we evaluated the activation and the role of autophagy in hMSC after inhibiting the expression of *MagT1* or *TRPM7* or culturing the cells under Mg deficient conditions.

## Results

### TRPM7 and MagT1 are overexpressed in hMSC induced to differentiate into osteoblasts

**Figure 1:**
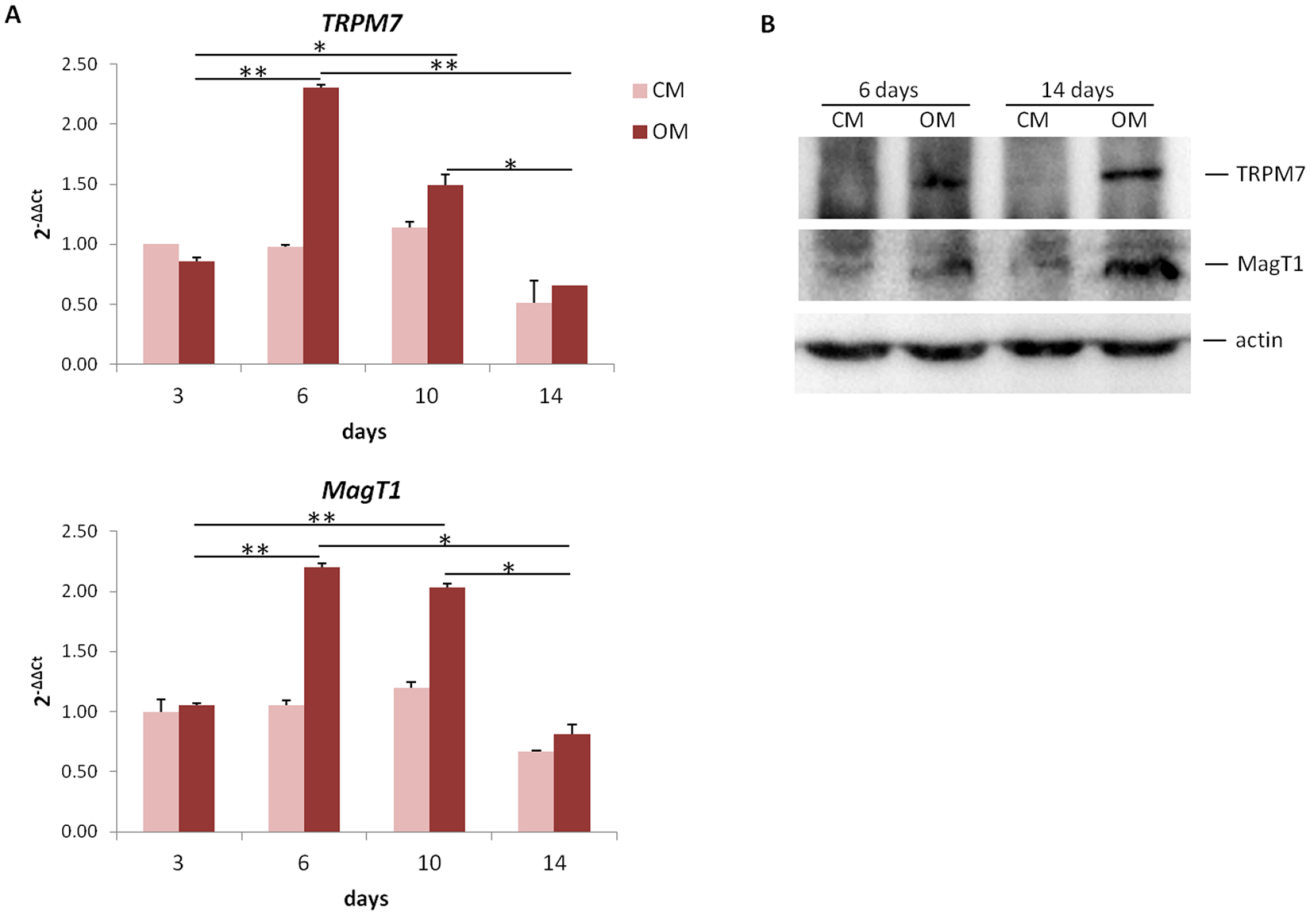
Osteogenic differentiation associates with the upregulation of TRPM7 and MagT1. (**A**) hMSC were cultured in OM or CM for 3, 6, 10 and 14 days. Real-Time PCR was performed on RNA extracted from hMSC using primers designed on *TRPM7* and *MagT1* sequence. (**B**) Western blot was performed on extracts from hMSC cultured in OM or CM for 6 and 14 days using antibodies against TRPM7 or MagT1. Actin was used as a control of loading. A representative blot is shown and quantification is provided in the Supplementary information (Fig. S1A).

Confluent cells were cultured for 3, 6, 10 and 14 days in an osteogenic medium containing vitamin D (OM) or in their culture medium (CM) as a control. By real-time PCR we demonstrate an overexpression of *TRPM7* and *MagT1* in cells exposed to OM for 6 and 10 days from the beginning of the experiment (Fig. 1A). Western blot shows that both TRPM7 and MagT1 are upregulated in hMSC exposed to the osteogenic medium for 6 and 14 days (Figs 1B and S1A,B). It is noteworthy that while the expression of *TRPM7* and *MagT1* drops at day 14, the protein levels remain elevated until the end of the experiment.

### siRNAs against *TRPM7* or *MagT1* boost the expression of osteogenic differentiation markers

**Figure 2:**
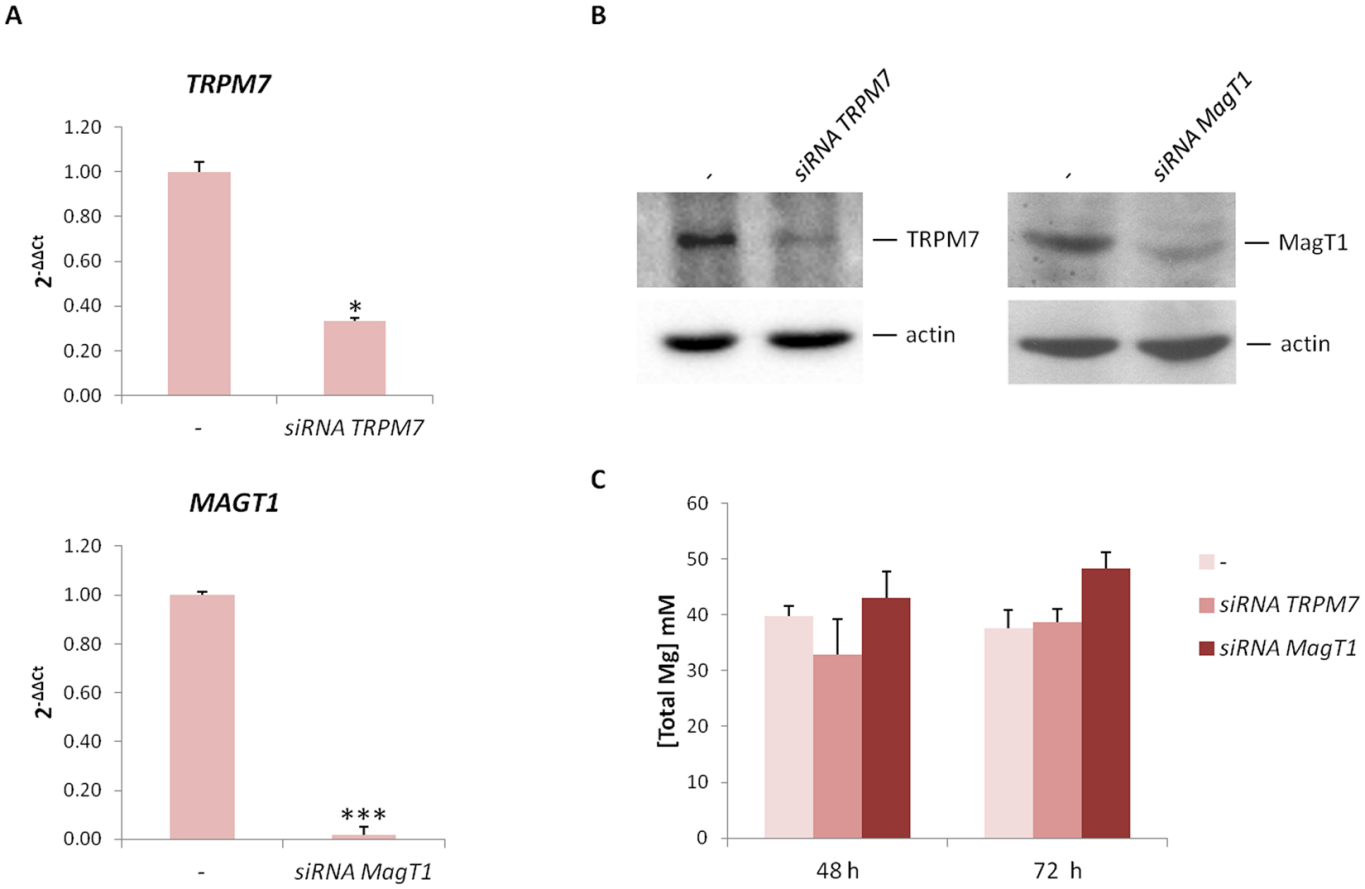
Specific siRNAs silence *TRPM7* or *MagT1* in hMSC. (**A**) After exposure to siRNA, hMSC were cultured in CM for 3 days. Real-Time PCR was performed on RNA extracted from hMSC using primers designed on the sequence of *TRPM7* and *MagT1*. The controls, indicated as -, were exposed to non-silencing, scrambled sequences. (**B**) Western blot was performed on extracts from hMSC after 3 days silencing. Antibodies against TRPM7 or MagT1 were used. Actin was used as a control of loading. A representative blot is shown and quantification is provided in the Supplementary information (Fig. S2A). (**C**) Total Mg was measured using the fluorescent chemosensor DCHQ5 as described.

To investigate the role of *TRPM7* and *MagT1* in the osteogenic differentiation of hMSC, we transiently transfected the cells with specific siRNAs for *TRPM7*, *MagT1* or with a non-silencing siRNA as a control (−) and then cultured hMSC in CM for 3 days. By real-time PCR *MagT1* expression resulted completely suppressed and *TRPM7* expression appeared dramatically reduced but not totally abrogated (Fig. 2A). Western blot shows the downregulation of the two proteins (Figs 2B and S2A,B). To get an overview about Mg homeostasis, we measured total intracellular Mg 48 and 72 h after transfection without finding any significant difference between cells silencing *TRPM7* or *MagT1* and relative controls (Fig. 2C).

**Figure 3:**
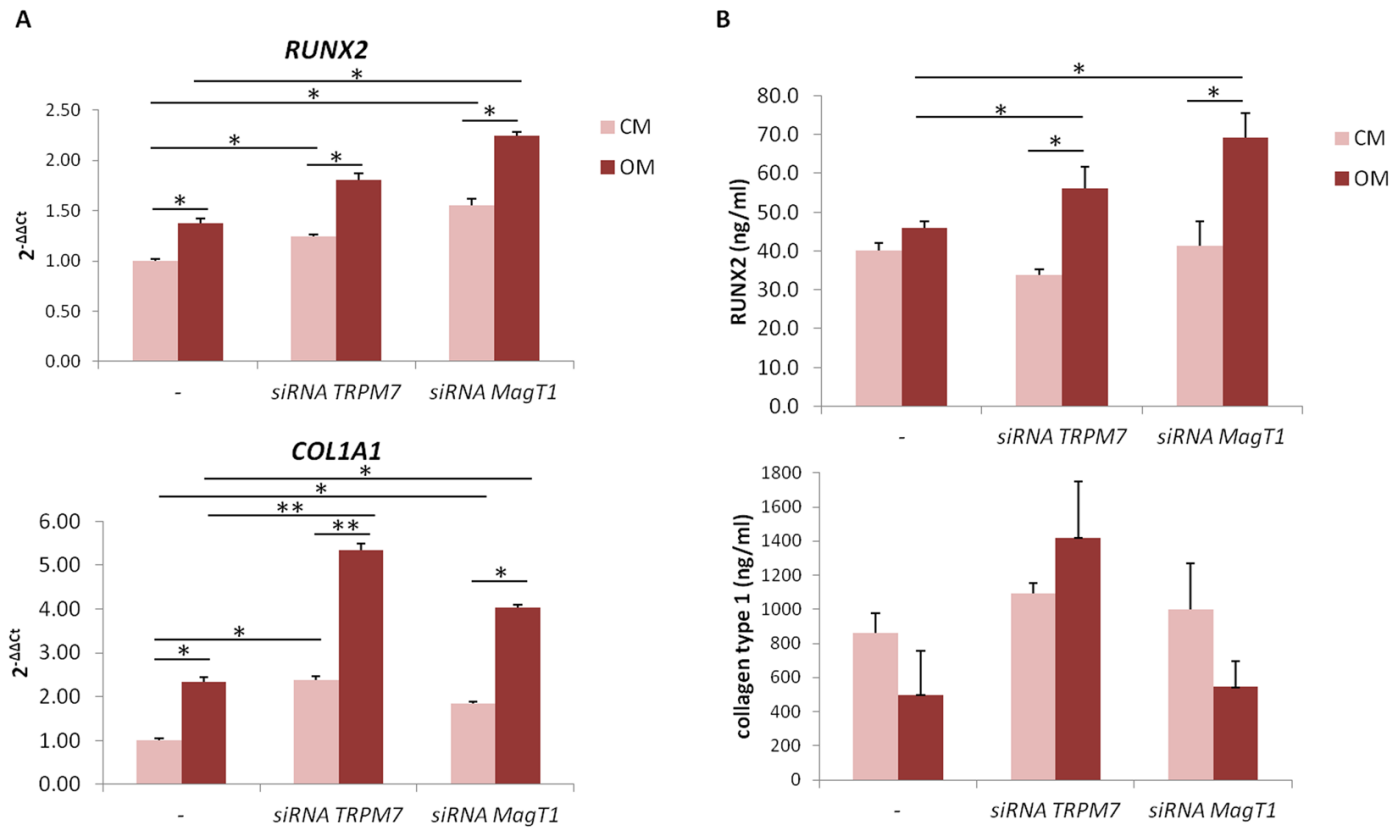
siRNAs targeting *TRPM7* or *MagT1* enhance osteogenic differentiation. (**A**) After exposure to siRNA, hMSC were cultured in CM or OM for 3 days. Real-Time PCR was performed on RNA extracted from hMSC using primers designed on *RUNX2* and *COL1A1* sequence. (**B**) ELISA for RUNX2 and collagen type 1 was conducted on extracts from hMSC cultured in CM or OM for 5 days. The controls, indicated as -, were exposed to non-silencing, scrambled sequences.

In siRNA transfected cells, we analyzed the expression of *RUNX2* and *COL1A1* and found it slightly upregulated even in the absence of the osteogenic cocktail (Fig. 3A). Moreover, cells downregulating *TRPM7* or *MagT1* maintained their sensitivity to the stimulatory effect of the osteogenic cocktail and upregulated both *RUNX2* and *COL1A1* more than controls (Fig. 3A). By ELISA, we demonstrate the significant increase of RUNX2 in hMSC downregulating *TRPM7* or *MagT1* after 5 days of osteogenic induction (Fig. 3B), while collagen type 1 is not induced probably because its accumulation represents a late event in osteoblastogenesis^15^.

**Figure 4:**
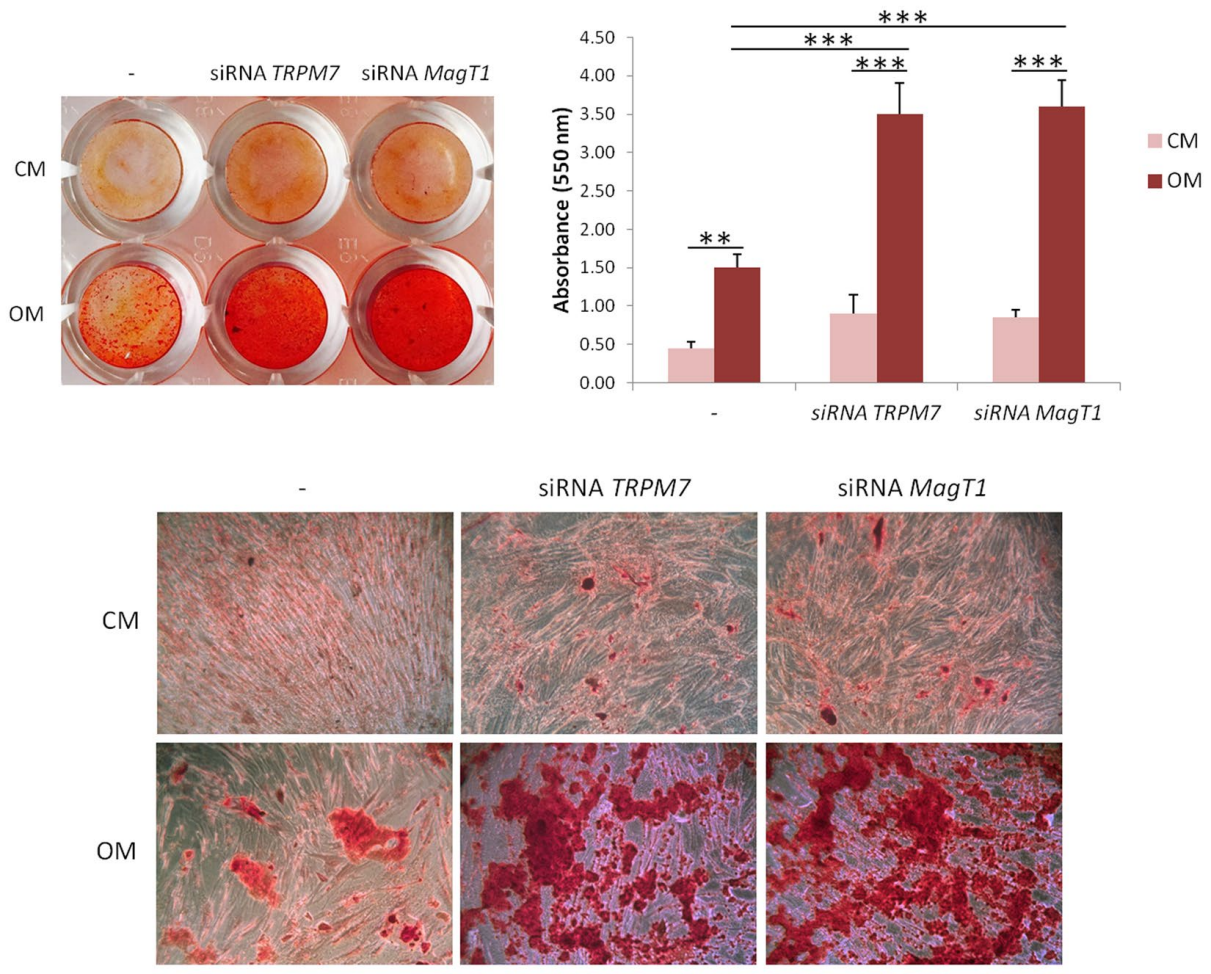
siRNAs targeting *MagT1* or *TRPM7* induce the deposition of calcium. Alizarin Red S staining was performed after exposure to CM or OM for 14 days. Whole well image (upper left panel) and photographs taken at 10X magnification (lower panel) are shown. After acid extraction the absorbance was measured at 550 nm (upper right panel).

Osteogenic differentiation of hMSC ultimately leads to the deposition of calcium in the extracellular matrix. We therefore evaluated calcium deposition by Alizarin Red S staining^16^ in silenced cells cultured in OM or CM for 14 days. In support to the results obtained by RT-PCR and ELISA, we detected some calcium deposits in siRNA transfected cells cultured in CM, but not in controls transfected with non silencing siRNAs (Fig. 4). Upon exposure to OM, siRNA transfected hMSC deposited much higher amounts of calcium phosphate crystals than non silenced cells.

### Autophagy is involved in accelerating osteogenesis in hMSC exposed to siRNAs against *TRPM7* or *MagT1*

**Figure 5:**
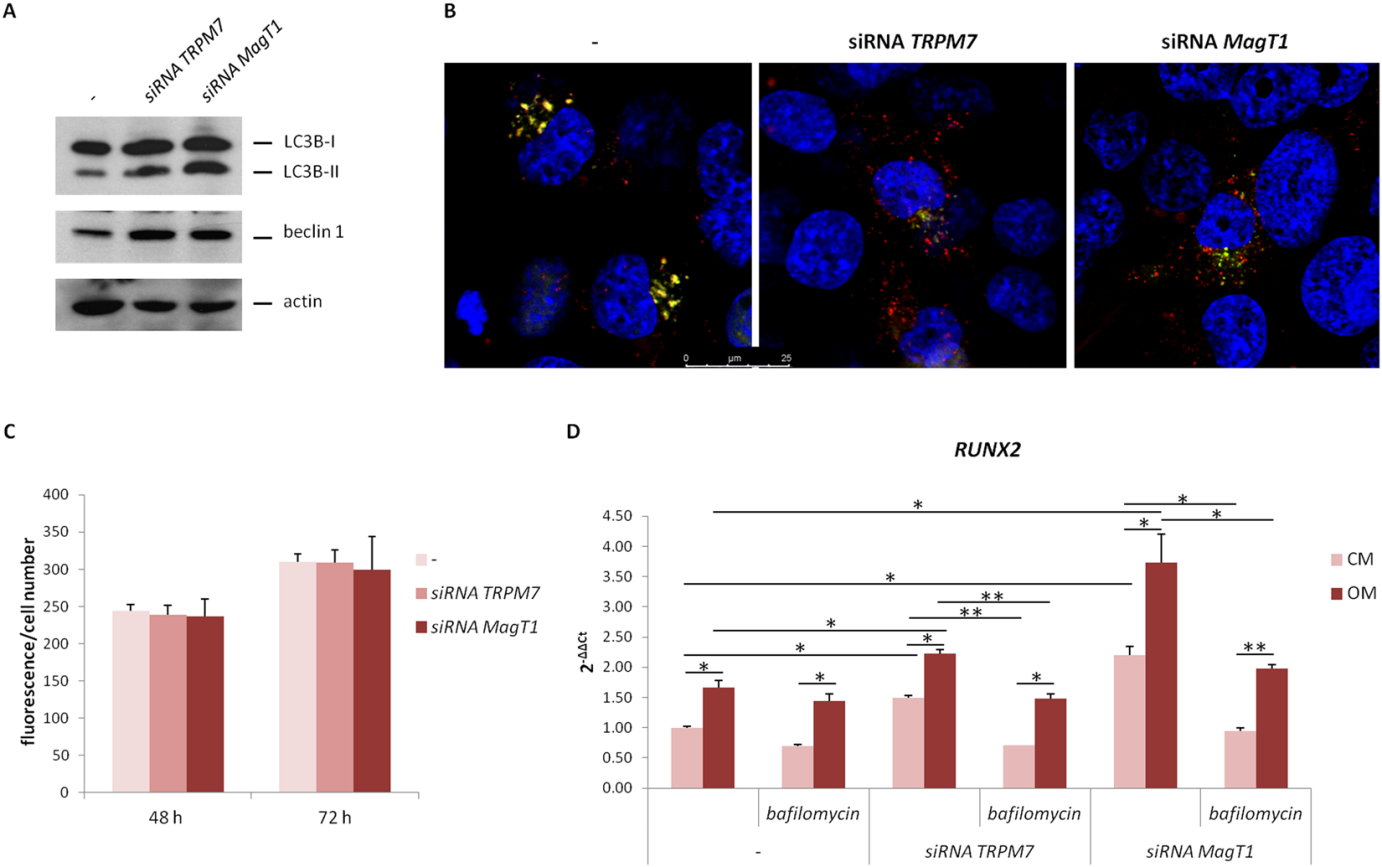
siRNAs targeting *TRPM7* or *MagT1* induce autophagy. hMSC were exposed to siRNAs targeting *TRPM7* or *MagT1*, or to non-silencing sequences (−), for 3 days. (**A**) The cells were lysed and Western blot was performed using antibodies against LC3B and beclin 1. Actin was used as a control of loading. A representative blot is shown and quantification is provided in the Supplementary information (Fig. S5A). (**B**) Autophagic flux was detected by Tandem fluorescent-tagged LC3 assay as described in the methods. (**C**) Intracellular free Ca was measured using Fura-2-AM as described. (**D**) Real-Time PCR was performed on RNA extracted from hMSC in CM or OM, treated or untreated with bafilomycin A1 (10 nM) for 3 days. Primers designed on *RUNX2* sequence were used.

Autophagy contributes to the osteogenic differentiation of hMSC^20^. In agreement with these findings, we show the conversion of microtubule-associated protein 1A/1B light-chain phosphatidylethanolamine conjugate (LC3B) to autophagosome-associated LC3B-II, which is the most widely used autophagosome marker^21^, in hMSC treated with siRNAs targeting *MagT1* or *TRPM7* for 3 days (Figs 5A and S5A,B). We also evaluated the total amounts of beclin 1, which contributes to the initiation of autophagosome formation by interacting with phosphatidylinositol 3-kinase^22^. Figures 5A and S5A,B show that beclin 1 is markedly induced in cells silencing *TRPM7* or *MagT1*.

To reinforce these results, we performed a Tandem fluorescent-tagged LC3 assay to assess autolysosome function. hMSC were transfected with the plasmid containing the sequence for the fusion protein LC3-GFP-RFP. Briefly, in the lysosome where the pH is low, the fluorescence of GFP is quenched, while that of RFP is stable. Formation of autophagosomes increases the number of GFP-positive/RFP-positive (yellow) vesicles, which become GFP-negative/RFP-positive (red) once fusion with lysosomes occurs. Figure 5B confirms the induction of autophagy in cells silencing *TRPM7* or *MagT1* as indicated by the increase of the number of vesicles and of autophagolysosomes in silenced cells *vs* controls.

Considering the complex role of free calcium in autophagy^23^, it is noteworthy that we did not detect any modulation of intracellular free Ca in silenced cells vs their controls (Fig. 5C).

Next we tested whether bafilomycin A1, which inhibits autophagy by preventing the acidification of endosomes and lysosomes, affects the expression of *RUNX2* in hMSC exposed to siRNA targeting *MagT1* or *TRPM7* for 3 days. Figure 5D shows that bafilomycin A1 prevented the increase of *RUN*X2 expression in siRNA treated cells, thus indicating that autophagy contributes to accelerating osteogenic differentiation in cells silencing *TRPM7* or *MagT1*.

### Low extracellular Mg enhances osteogenic differentiation of hMSC by activating autophagy

**Figure 6:**
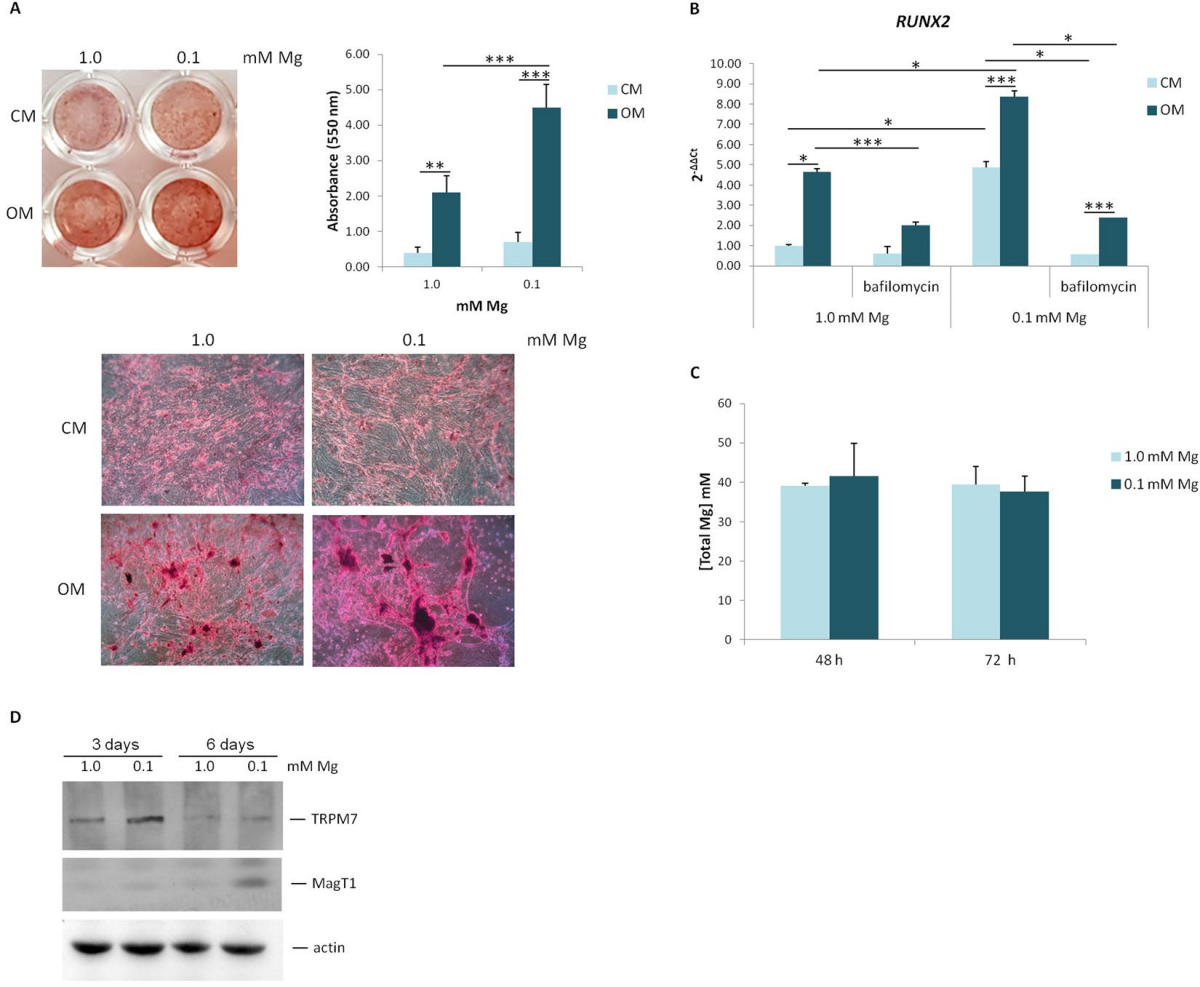
Mg deficiency accelerates osteogenic differentiation. (**A**) Alizarin Red S staining was performed on hMSC cultured in 0.1 or 1.0 mM Mg with or without the osteogenic cocktail for 14 days. Whole well image (upper left panel) and photographs taken at 10X magnification (lower panel) are shown. Absorbance was measured at 550 nm after acid extraction (upper right panel). (**B**) Real-Time PCR was performed on RNA extracted from hMSC cultured in 1.0 or 0.1 mM Mg, additioned or not with the osteogenic cocktail, treated or untreated with bafilomycin A1 (10 nM) for 3 days. Primers designed on *RUNX2* sequence were used. (**C**) Total Mg was measured using the fluorescent chemosensor DCHQ5 as described. (**D**) Western blot was performed on extracts from hMSC cultured in 1.0 or 0.1 mM Mg for 3 and 6 days using antibodies against TRPM7 or MagT1. Actin was used as a control of loading. A representative blot is shown and quantification is provided in the Supplementary information (Fig. S6A).

We asked whether Mg deprivation has an effect on hMSC differentiation and found that culture in 0.1 mM Mg for 14 days enhances Ca deposition in the extracellular matrix as detected by Alizarin Red S staining (Fig. 6A). Accordingly, the expression of *RUN*X2 is higher in Mg deficient cells versus their controls both in the presence and in the absence of osteogenic stimuli (Fig. 6B).

No differences were observed in the content of total intracellular Mg in hMSC maintained in Mg deficient medium vs controls (Fig. 6C). Moreover, western blot shows that TRPM7 is upregulated after 3 days and MagT1 after 6 days of culture in medium containing 0.1 mM Mg (Figs 6D and S6A,B).

**Figure 7:**
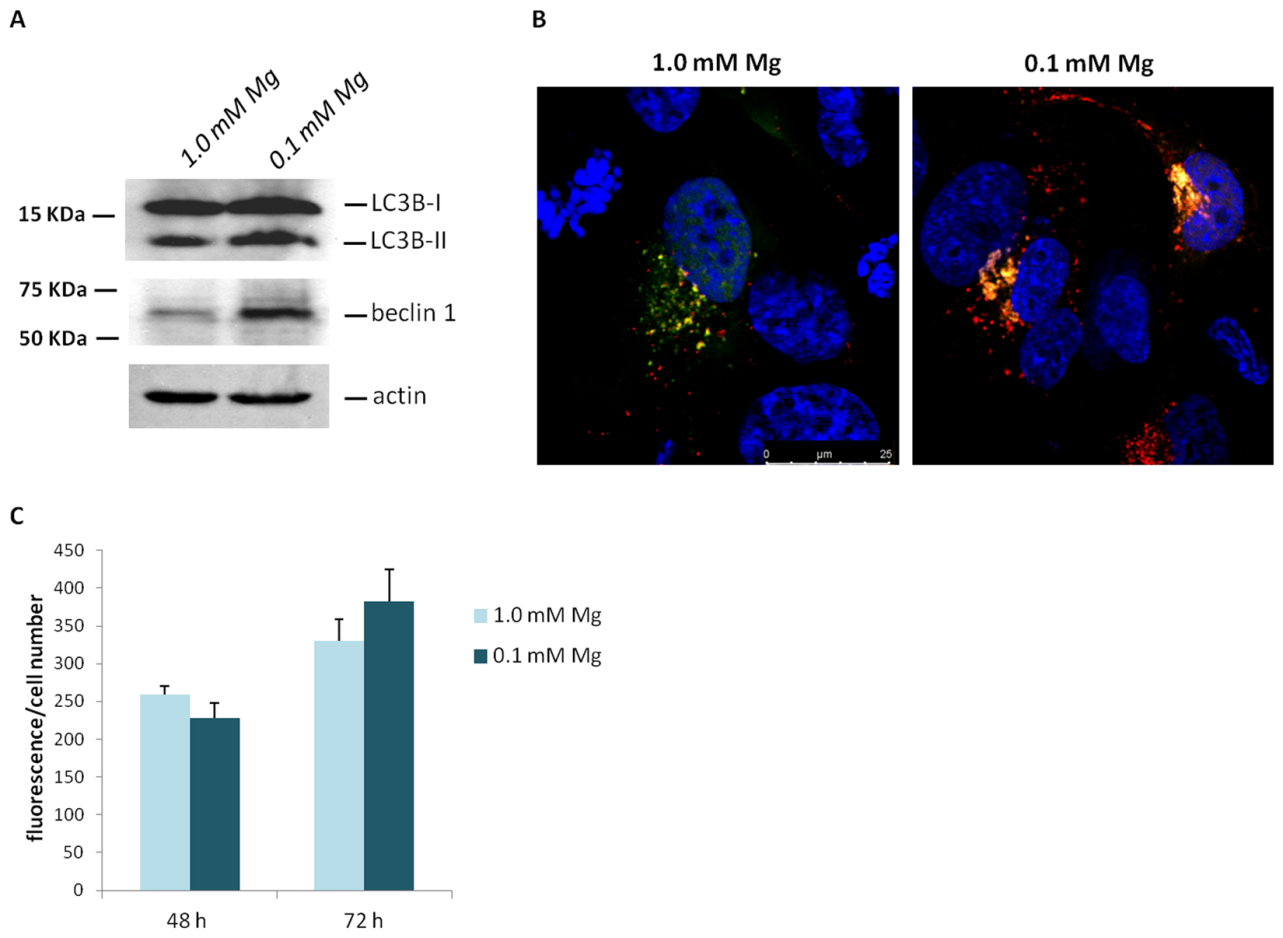
Mg deficiency induces autophagy. (**A**) hMSC were cultured in 0.1 or 1.0 mM Mg. After 3 days the cells were lysed and Western blot was performed using antibodies against LC3B and beclin 1. Actin was used as a control of loading. A representative blot is shown and quantification is provided in the Supplementary information (Fig. S7A). (**B**) Autophagic flux was detected by Tandem fluorescent-tagged LC3 assay as described in the methods. (**C**) Intracellular free Ca was measured using Fura-2-AM as described.

Next, we demonstrate that culture in low extracellular Mg (0.1 mM Mg) stimulates the conversion of LC3B to LC3B-II and upregulates beclin 1 in our experimental model (Figs 7A and S7A,B). These results were confirmed by Tandem fluorescent-tagged LC3 assay described above which shows an increase of the number of vesicles and of autophagolysosomes in Mg deficient hMSC (Fig. 7B). Figure 7C demonstrates no variation of intracellular free Ca between cells cultured in 0.1 or 1.0 mM Mg medium.

The contribution of autophagy to osteogenic differentiation is supported by the fact that bafilomycin A1 reduced the expression of *RUN*X2 in cells cultured in low (0.1 mM) or normal (1.0 mM) extracellular Mg for 3 days (Fig. 6B).

To the best of our knowledge this is the first evidence of a link between Mg, its transporters and autophagy.

## Discussion

Mg is required for every biological process not only because it is necessary for the function of hundreds of enzymes but also because it maintains the active conformation of macromolecules, regulates second messengers, various transporters and ion channels^12^. Therefore, it is not surprising that the amounts of intracellular Mg are balanced by a coordinated interplay among channels and transporters mediating Mg influx, exchangers regulating its efflux, and Mg shuffling from organelles to cytosol and viceversa^24^.

About 60% of total Mg is stored in the bone and a tight control of magnesium homeostasis is crucial for bone health^13^. A key event in bone formation is the differentiation of MSC into osteoblasts, a process which involves Mg and its transporters^5–7,11,25^. It has recently been reported that TRPM7, which is important in skeletogenesis^26^, acts as a mechanotransducer in murine MSC and commits their fate towards osteogenic differentiation in response to mechanical stimulation^5^. Also MagT1^11^ and the Na/Mg exchanger SLC41A^27^ contribute to osteoblastogenesis^28^.

We focused our studies on TRPM7 and MagT1, both contributing to Mg transport into the cells^12^, in a model of hMSC induced to differentiate into osteoblasts. We show the overexpression of *TRPM7* and *MagT1* with a peak after 6 days of osteogenic induction and a gradual return to control levels within 14 days. The increase of the transcripts is paralleled by the upregulation of TRPM7 and MagT1 at the protein level, which is retained up to day 14 from osteogenic induction. We hypothesize that post-translational mechanisms might be implicated in maintaining high the amounts of TRPM7 and MagT1 until hMSC reach full differentiation. While further studies are required, it is noteworthy that our results with MagT1 are in agreement with a report showing the increase of MagT1 in rat MSC exposed to an osteogenic medium^11^.

To unravel the function of TRPM7 and MagT1 in hMSC, we utilized specific siRNAs and demonstrate that downregulating *TRPM7* or *MagT1* accelerates hMSC osteogenic differentiation. Our observation in hMSC silencing *MagT1* is in disagreement with previous results in rat MSC showing that knocking down *MagT1* inhibits osteogenic differentiation^11^. This discordance could be ascribed either to the different behavior of cells of different species or to the different protocols used to induce differentiation. While we used vitamin D to induce osteogenic differentiation, Zheng *et al*. used dexamethasone which has been shown to modulate TRPM7 in some tissues^29^.

Since i) MagT1 and TRPM7 are upregulated in hMSC differentiation, and ii) siRNAs targeting *TRPM7* or *MagT1* hasten the expression of osteogenic markers as well as Ca deposition, we hypothesize that these transporters contribute to coordinate hMSC response to osteogenic stimuli by preventing excessive osteoblastogenesis. In this light, TRPM7 and MagT1 might be considered as sensors-controllers of the extent of hMSC osteogenic differentiation.

Silencing *TRPM7* in different cell types, among which colon carcinoma cells, thymocytes and human endothelial cells, does not influence intracellular Mg content^30–32^, while knocking down *MagT1* in mammalian cell lines lowered the levels of intracellular Mg^33^. We did not detect differences in intracellular Mg content in hMSC silencing *TRPM7* or *MagT1*, suggesting that either TRPM7 or MagT1 is sufficient to maintain intracellular Mg homeostasis in hMSC. Considering that many different Mg transporters have been described and account for Mg balance^24^, it is feasible to propose that also other proteins contribute to maintain Mg intracellular concentrations.

A connection between bone health and autophagy has been described^17–19^. It is noteworthy that autophagy is activated in the early phases of hMSC differentiation^20^. It is also interesting that resting quiescent hMSC accumulate autophagosomes^34^, which indicates that the cells are ready to react to different stimuli by activating autophagy. Moreover, autophagy is induced in embryonic stem cells early during differentiation^35^ and in somatic cells reprogrammed to induced pluripotent stem cells^36^. We suggest that siRNA targeting *TRPM7* or *MagT1* boosts the expression of markers of osteogenic differentiation, at least in part, by activating autophagy, which removes unnecessary organelles, generates substrates to supply energy and recycles macromolecular blocks to drive cell differentiation. Accordingly, bafilomycin A1, an inhibitor of autophagy, prevents the acceleration of osteogenic differentiation in our experimental model. Ca does not seem to be implicated in triggering autophagy in cells silencing *TRPM7* or *MagT1* as well as in hMSC cultured in low extracellular Mg. To this purpose, it is noteworthy that early after exposure to a Mg deficient medium, TRPM7 increases, while later, when TRPM7 declines to baseline levels, MagT1 is upregulated. We hypothesize that the sequential upregulation of the two proteins allows to maintain unaltered the concentrations of intracellular Mg. Moreover, Mg deficiency mimics the effects of silencing *TRPM7* or *MagT1* because it accelerates the osteogenic differentiation of hMSC^25^ and this happens in association with the trigger of autophagy without any detectable increase of free Ca.

Apart from transporting Mg, TRPM7 and MagT1 serve other functions. MagT1 contributes to the oligosaccharyltransferase complex, thus being involved in the glycosylation of various proteins^37^. It should be noted that the components of the extracellular matrix are glycosylated and this is important for bone formation and remodeling^38^. Moreover, it has been proposed that MagT1 might regulate Mg transport by glycosylating Mg transporters^24^. TRPM7 possesses a kinase domain and we cannot rule out the possibility that silencing *TRPM7* might affect the phosphorylation of specific substrates, some of which have been identified^24^. Of interest, one of the protein phosphorylated by TRPM7 is annexin 1, a Ca-regulated phospholipid binding protein implicated in the regulation of cell growth, apoptosis^39,40^ and also in osteogenic differentiation^41^. On these bases, downregulating *TRPM7* and/or *MagT1* might impact on the post-translational modification of proteins.

In conclusion, we demonstrate that MagT1 and TRPM7 as well as Mg deficiency contribute to the regulation of hMSC osteogenic differentiation partly by modulating autophagy in a Ca-independent fashion.

## Methods

### Culture of hMSC

hMSC were isolated from adult human bone marrow withdrawn from bilateral punctures of the posterior iliac crests of healthy male volunteers, after obtaining informed consent from all the subjects at the Policlinico in Milano, in compliance with the Helsinki declaration, according to institutional guidelines and regulations of the Ethical Committee of “IRCCS Policlinico” Milano. These cells were used in previous studies^16,25^.

The cells were cultured at 37 °C and 5% CO_2_ in Dulbecco’s Modified Eagle’s Medium containing 10% fetal bovine serum and 2 mM glutamine (culture medium, CM). All the reagents for cell culture were from Sigma-Aldrich. The cells were used between passage 2 and 6.

To obtain a transient downregulation of MagT1 and TRPM7, we utilized the stealth siRNAs developed by Qiagen for *TRPM7* and Invitrogen for *MagT1*. siRNAs targeting *TRPM7* were transfected using HiPerFect Transfection Reagent (Qiagen) while for siRNAs targeting MagT1 we used Lipofectamine RNAiMAX (Thermo Fisher Scientific). Non-silencing, scrambled sequences were used as controls (−)^42^. Viable cells were counted using a cell counter (Logos Biosystems).

In some experiments, hMSC were cultured in Mg-free MEM (Invitrogen, Thermo Fisher Scientific) or Mg-free MEM supplemented with MgSO_4_ (Sigma-Aldrich) to reach the physiological concentration, i.e. 1 mM.

To induce osteogenic differentiation, hMSC were seeded in 6-well or 96-well plates. Once the cells were confluent, an osteogenic induction cocktail was added to the medium (osteogenic medium, OM). The osteogenic cocktail contains 2 × 10^−8^ M 1α,25-Dihydroxyvitamin D3, 10 mM β-glycerolphosphate and 0.05 mM ascorbic acid (Sigma-Aldrich)^16^. To analyze Ca deposition by hMSC, the cells were rinsed with PBS, fixed (70% ethanol, 1 h) and stained for 10 min with 2% Alizarin Red S (pH 4.2, Sigma-Aldrich)^43^. Alizarin Red S staining was released from the cell matrix by incubation in 10% cetylpyridinium chloride in 10 mM sodium phosphate (pH 7.0), for 15 min and the absorbance measured at 550 nm. The experiment was repeated three times in triplicate. Photographs were taken at 10X magnification.

### Real-Time PCR

Total RNA was extracted by the PureLink RNA Mini kit (Thermo Fisher Scientific). Single-stranded cDNA was synthesized from 0.2 µg RNA in a 20 µL final volume using High Capacity cDNA Reverse Transcription Kit, with RNase inhibitor (Thermo Fisher Scientific) according to the manufacturer’s instructions. Real-time PCR was performed using the 7500 FAST Real Time PCR System instrument using TaqMan Gene Expression Assays (Life Technologies, Thermo Fisher Scientific): Hs00231692_m1 (*RUNX2*), Hs00164004_m1 (*COL1A1*), Hs00918928_g1 (*TRPM7*), Hs00997540_m1 (*MagT1*).

The housekeeping gene *GAPDH* (Hs99999905_m1) was used as an internal reference gene. Relative changes in gene expression were analyzed by the 2^−ΔΔCt^ method^16^. The experiments were repeated three times in triplicate.

### Western blot analysis

Western blot was performed on hMSC induced to differentiate into osteoblasts, exposed to siRNAs targeting *TRPM7* and/or *MagT1* or cultured in medium containing 0.1 mM Mg. After lysis, samples (80 µg/lane) were separated on SDS-polyacrylamide gel, transferred to nitrocellulose sheets at 150 mA for 16 h, and probed with antibodies against TRPM7 (Bethyl), MagT1 (Abcam), LC3B, beclin 1 (Cell Signalling Technology) and actin (Santa Cruz Biotechnology). Secondary antibodies were labelled with horseradish peroxidase (GE healthcare). The ECL Western Blotting Substrate (Thermo Fisher Scientific) was used to detect immunoreactive proteins. The western blots were repeated at least three times and a representative blot is shown. Densitometry was performed using ImageJ software and results are shown as the mean ± standard deviation.

### Tandem fluorescent-tagged LC3 assay

The Tandem fluorescent-tagged LC3 assay, which is based on the different pH stability of two fluorescent proteins, allows to monitor autophagic flux and provides information about the number of vesicles and/or the progression to the late phase of autophagy^44^. Briefly, hMSC were transfected with a plasmid containing the sequence coding for the LC3 protein fused with two fluorophores, specifically RFP (red) and GFP (green) using lipofectamine 2000 for 4 h. Twenty-four hours post transfection hMSC were silenced for *TRPM7* or *MagT1* as described above. 48 h later the cells were fixed and analysed by confocal microscopy (LEICA SP8, magnification 40x). The early phase, characterized by phagosome formation, is detectable through the visualization of vesicles containing the two fluorophores (yellow fluorescence). The late phase, characterized by the fusion of the phagosome with the lysosome and the formation of the phagolysosome, is characterized by the cleavage of the GFP fragment, which is sensitive to the acidic pH of the lysosome, so that only RFP signal is detected (red fluorescence).

### Quantification of intracellular Mg and Ca

Total Mg content was measured on sonicated hMSC using the fluorescent chemosensor DCHQ5 (kindly donated by Prof. S. Iotti, Università di Bologna) as described^31,32^. Fluorescence intensities were acquired at 510 nm. Mg concentrations of the samples were obtained by the interpolation of their fluorescence with the standard curve performed using MgSO_4_.

To quantify free Ca, Fura-2/AM (10 μM) was added to the culture medium for 60 min. Then the cells were washed with a buffer containing NaCl 125 mM, KCl 5 mM, MgSO4 1.2 mM, CaCl_2_ 2 mM, glucose 6 mM, Hepes-NaOH buffer 25 mM (pH 7.4), removed by trypsinization, and suspended in the aforementioned buffer. Fluorescence intensities were acquired using a spectrophotometer with excitation wavelengths of 360 nm and emission at 450 nm^45^. Results were normalized on cell number.

### ELISA

hMSC were exposed to CM or OM for 5 days. For the quantitative determination of RUNX2 and collagen type 1, Cusabio ELISA kit were used according to the manufacturer’s instructions. ELISAs were performed at least three times, and each sample was measured in triplicate. Data are shown as the mean ± standard deviation.

### Statistical analysis

Statistical significance was determined using Student’s t test and set as following: *P < 0.05, **P < 0.01, ***P < 0.001.

## Electronic supplementary material

Supplementary Information
