# Supplementary material for: TRPM7 and MagT1 in the osteogenic differentiation of human mesenchymal stem cells in vitro
Source: Sci Rep. 2018 Nov 1;8:16195. doi: 10.1038/s41598-018-34324-8 (PMC6212439; doi:10.1038/s41598-018-34324-8)

TRPM7 and MagT1 in the osteogenic differentiation of human mesenchymal stem cells

Sara Castiglioni, Valentina Romeo, Laura Locatelli, Alessandra Cazzaniga and Jeanette AM Maier

Supplementary Figures

Figure S1A

Densitometric analysis was performed by the ImageJ software and TRPM7 or MagT1/actin ratio was calculated on three separate experiments.

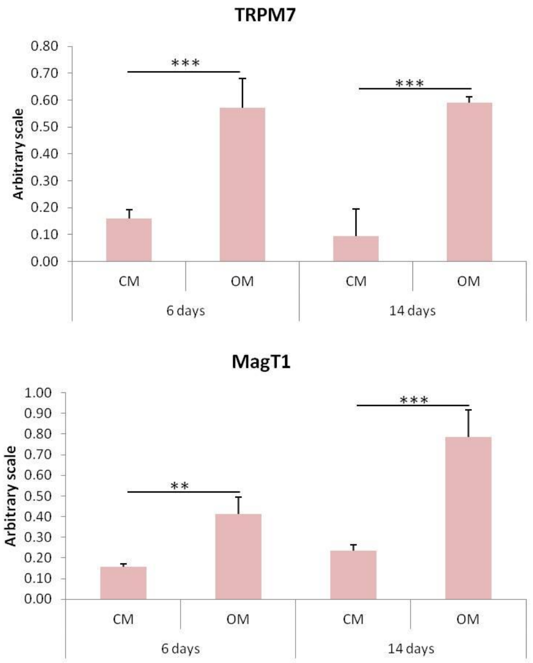

Figure S1B

Full-length blots performed on extracts from hMSC cultured in OM or CM for 6 or 14 days using antibodies against TRPM7 or MagT1. Actin was used as a control of loading.

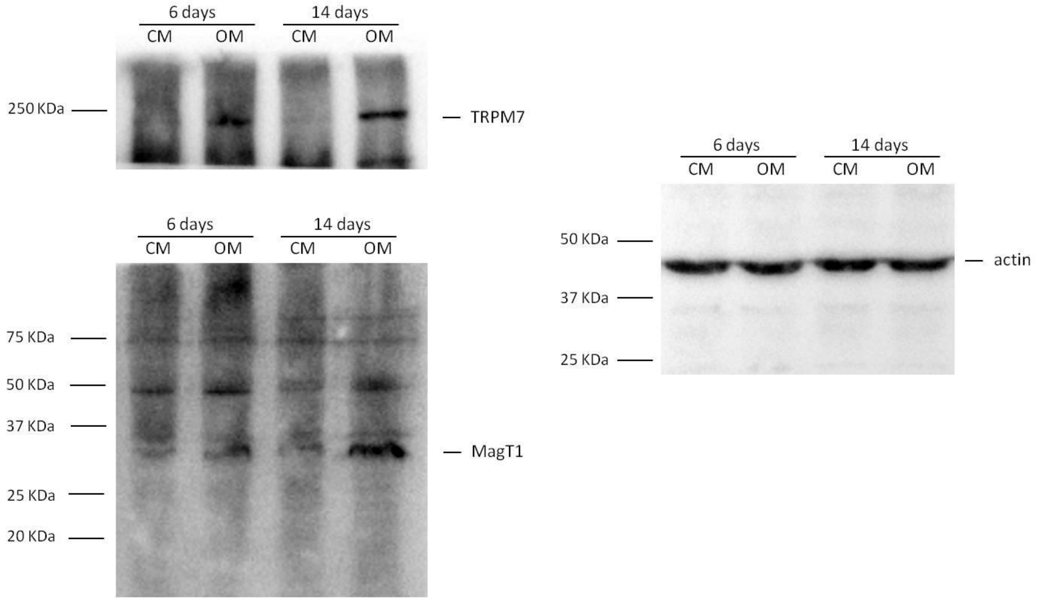

Figure S2A

Densitometric analysis was performed by the ImageJ software and TRPM7 or MagT1/actin ratio was calculated on three separate experiments.

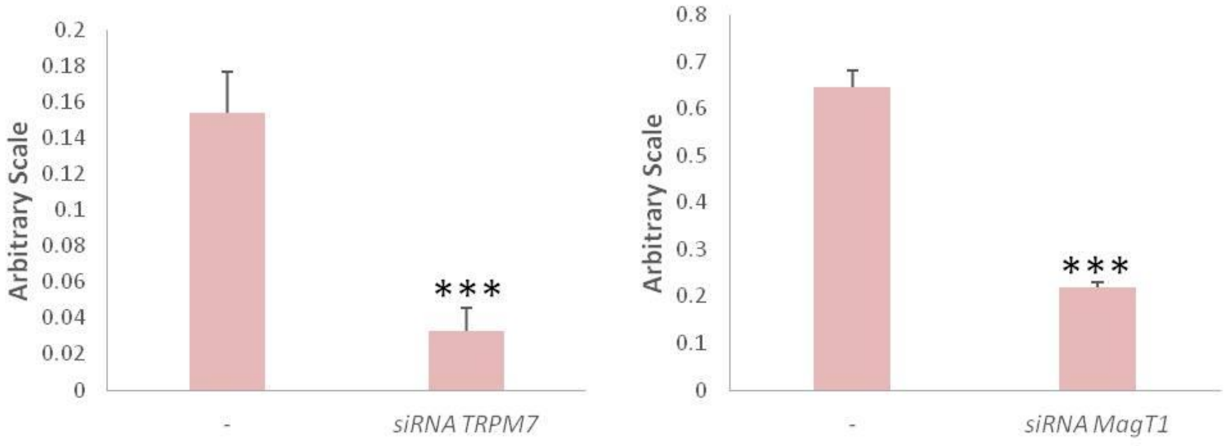

Figure S2B

Full-length blots performed on extracts from hMSC transfected with siRNAs targeting *TRPM7*, *MagT1* or non silencing sequences (-) using antibodies against TRPM7 and MagT1. Actin was used as a control of loading.

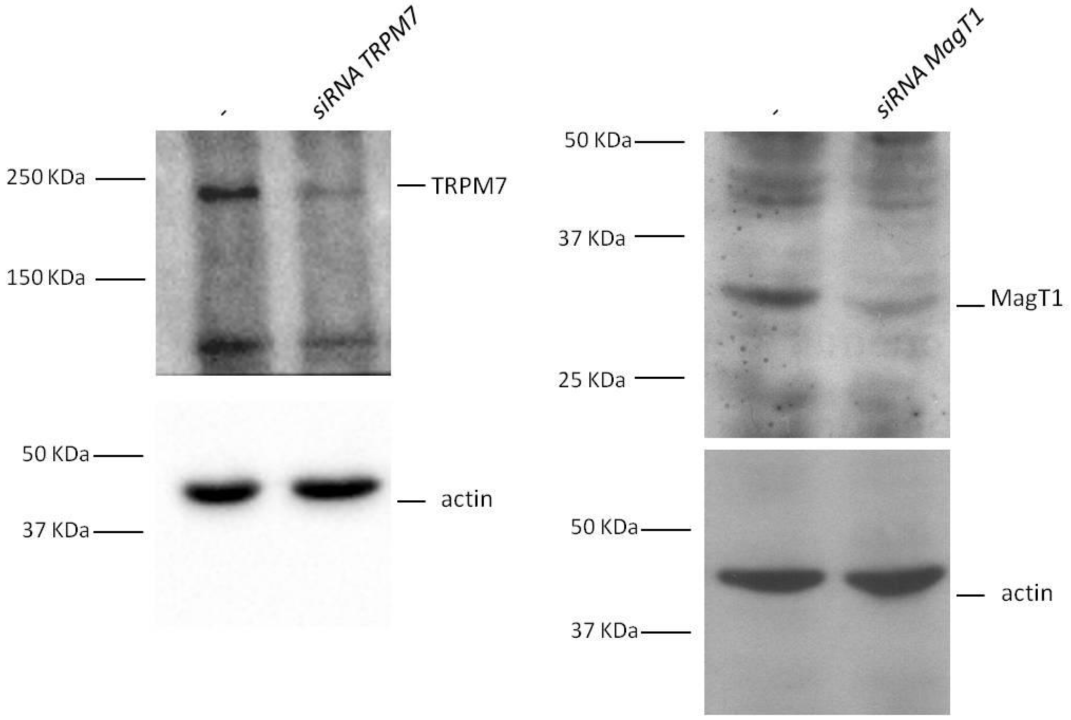

Figure S5A

Densitometric analysis was performed by the ImageJ software and LC3B-II or beclin 1/actin ratio was calculated on three separate experiments.

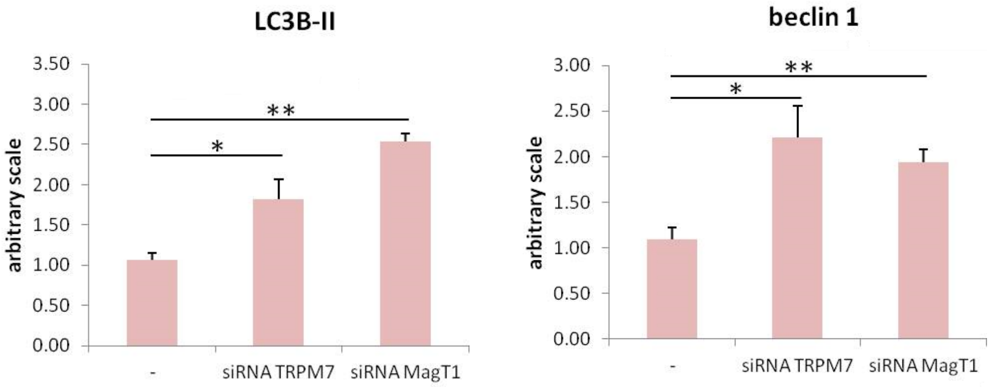

Figure S5B

Full-length blots performed on extracts from hMSC transfected with siRNAs targeting *TRPM7*, *MagT1* or non silencing sequences (-) using antibodies against LC3B and beclin 1. Actin was used as a control of loading.

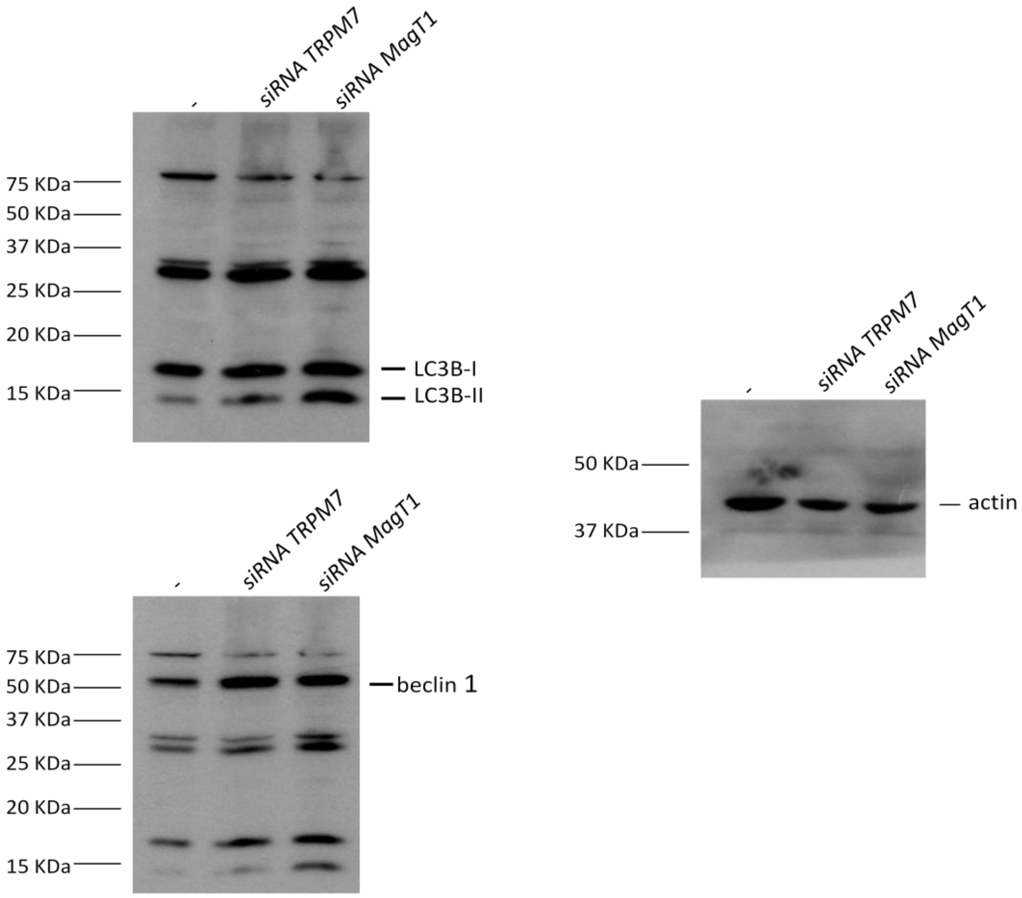

Figure S6A

Densitometric analysis was performed by the ImageJ software and TRPM7 or MagT1/actin ratio was calculated on three separate experiments.

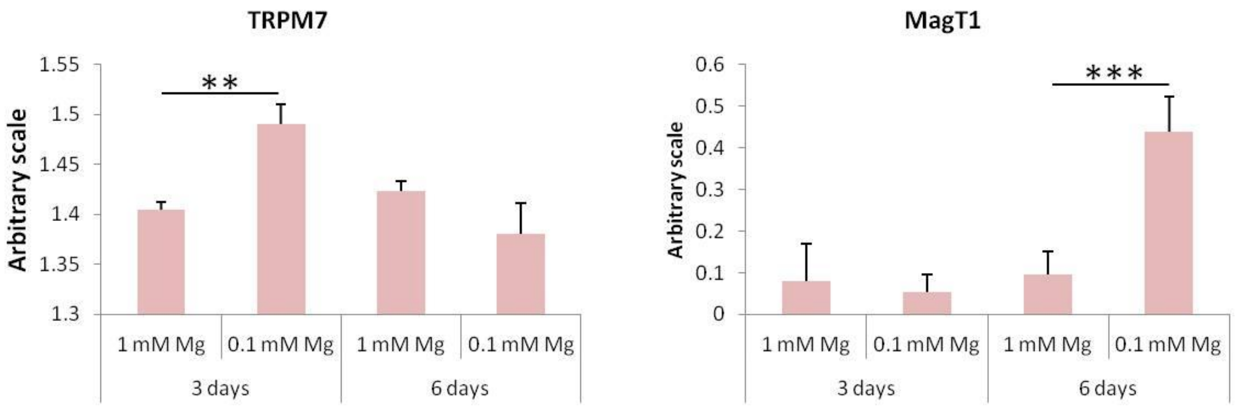

Figure S6B

Full-length blots performed on extracts from hMSC cultured in 1.0 or 0.1 mM Mg for 3 or 6 days using antibodies against TRPM7 or MagT1. Actin was used as a control of loading.

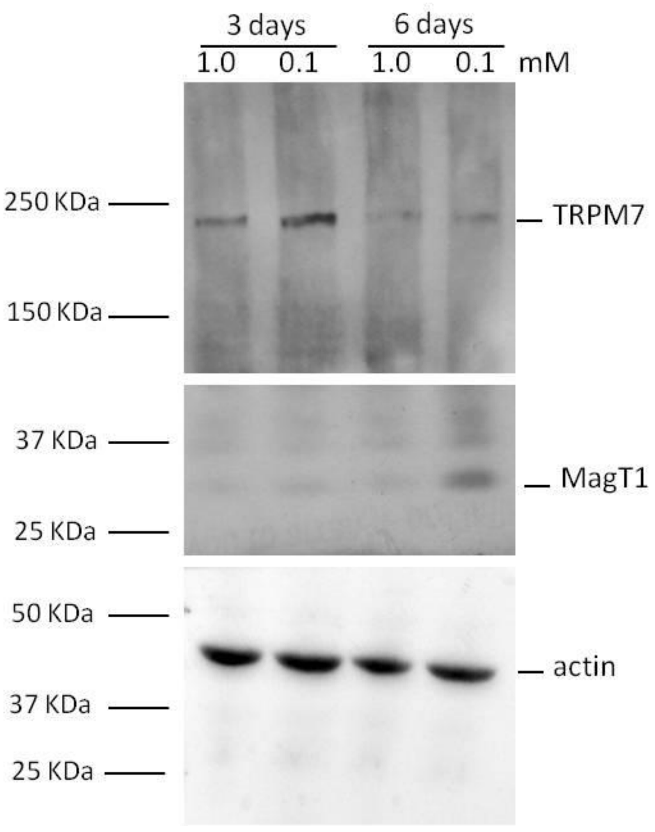

Figure S7A

Densitometric analysis was performed by the ImageJ software and LC3B-II or beclin 1/actin ratio was calculated on three separate experiments.

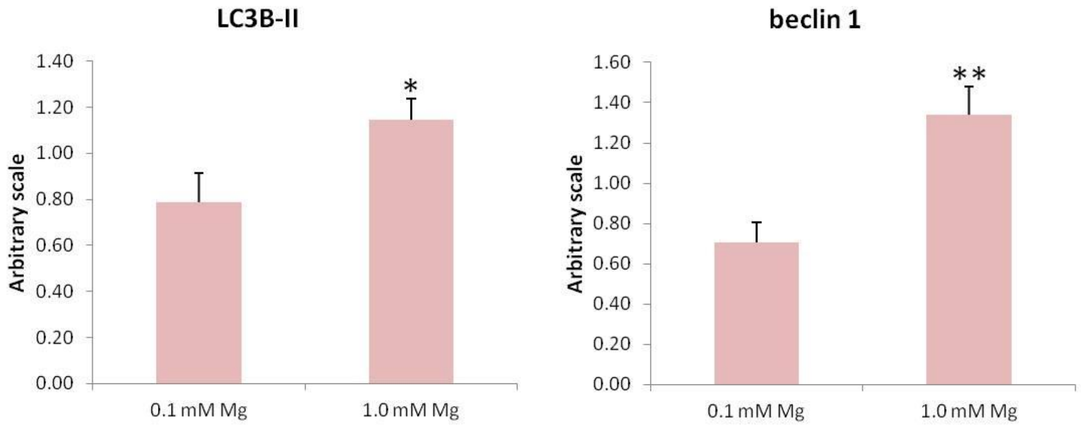

Figure S7B

Full-length blots performed on extracts from hMSC cultured in 0.1 or 1.0 mM magnesium using antibodies against LC3B and beclin 1. Actin was used as a control of loading.

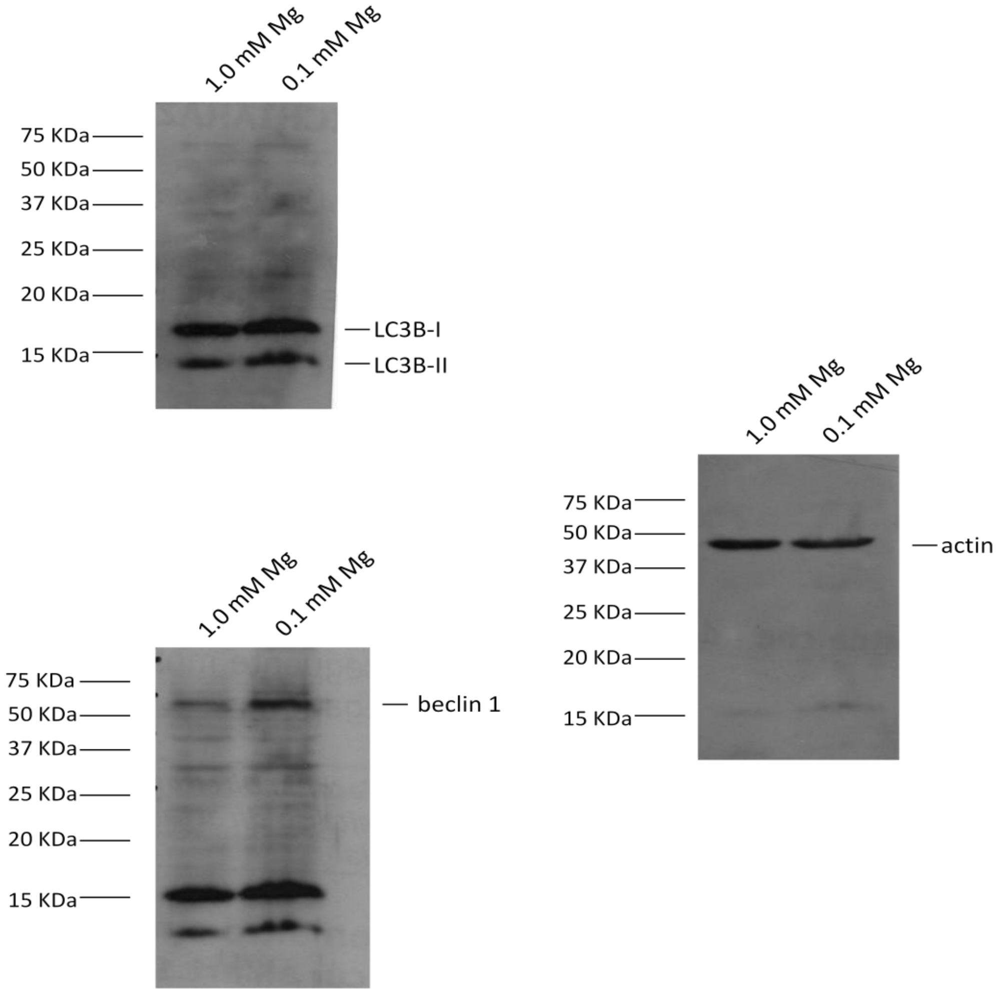

Supplement: Supplementary file 1 — Supplementary Information [file 41598_2018_34324_MOESM1_ESM.pdf]
